# Comprehensive Identification of mRNA-Binding Proteins of *Leishmania donovani* by Interactome Capture

**DOI:** 10.1371/journal.pone.0170068

**Published:** 2017-01-30

**Authors:** Devki Nandan, Sneha A. Thomas, Anne Nguyen, Kyung-Mee Moon, Leonard J. Foster, Neil E. Reiner

**Affiliations:** 1 Departments of Medicine, University of British Columbia, Vancouver, BC, Canada; 2 University of British Columbia, Centre for High-Throughput Biology and Department of Biochemistry & Molecular Biology, Vancouver, BC, Canada; 3 Department of Microbiology and Immunology, University of British Columbia, Vancouver, BC, Canada; John Curtin School of Medical Research, AUSTRALIA

## Abstract

Leishmania are unicellular eukaryotes responsible for leishmaniasis in humans. Like other trypanosomatids, leishmania regulate protein coding gene expression almost exclusively at the post-transcriptional level with the help of RNA binding proteins (RBPs). Due to the presence of polycystronic transcription units, leishmania do not regulate RNA polymerase II-dependent transcription initiation. Recent evidence suggests that the main control points in gene expression are mRNA degradation and translation. Protein-RNA interactions are involved in every aspect of RNA biology, such as mRNA splicing, polyadenylation, localization, degradation, and translation. A detailed picture of these interactions would likely prove to be highly informative in understanding leishmania biology and virulence. We developed a strategy involving covalent UV cross-linking of RBPs to mRNA *in vivo*, followed by interactome capture using oligo(dT) magnetic beads to define comprehensively the mRNA interactome of growing *L*. *donovani* amastigotes. The protein mass spectrometry analysis of captured proteins identified 79 mRNA interacting proteins which withstood very stringent washing conditions. Strikingly, we found that 49 of these mRNA interacting proteins had no orthologs or homologs in the human genome. Consequently, these may represent high quality candidates for selective drug targeting leading to novel therapeutics. These results show that this unbiased, systematic strategy has the promise to be applicable to study the mRNA interactome during various biological settings such as metabolic changes, stress (low pH environment, oxidative stress and nutrient deprivation) or drug treatment.

## Introduction

Regulation of gene expression requires an array of coordinated mechanisms that are used by cells to modulate the production of specific gene products. In eukaryotic cells, gene regulation starts at the level of transcription initiation, followed by regulation at the post-transcriptional level. Nascent mRNAs are subject to regulation via RNA processing, transport, localization, translation and degradation. During biosynthesis and after completion of transcription, nascent mRNAs associate with RNA binding proteins to form messenger ribonucleoprotein (mRNP) complexes that regulate most aspects of mRNA metabolism and function. In their dynamic association with mRNP complexes, a repertoire of RBPs plays critical roles in regulating RNA processing, transport, localization, translation and degradation. In fact, there is evidence for the existence of an extensive network of RBPs, which through combinatorial binding affects the post-transcriptional fate of every mRNA in the cell [1–4]. It is becoming increasingly clear that post-transcriptional processes play equally important roles in the output of gene products when compared to transcriptional control. Notably, *Leishmania* is one genus that has evolved to bypass the regulation of gene expression at the transcription initiation stage [5].

Leishmania are the causative agents of leishmaniases in humans. Transmitted by sand flies, these infections are responsible for severe morbidity and mortality, worldwide. To date, there are no effective vaccines against these parasites. Moreover, currently available drugs have significant toxicities and over time, these organisms are known to develop drug resistance. Leishmania have a digenetic life-cycle alternating between their mammalian hosts (amastigote stage) and their sandfly vectors (promastigote stage). Adaptation to distinct environments requires activation of specific gene networks. As described above, most eukaryotes activate gene networks by transcriptional mechanisms singularly, or in parallel with post-transcriptional regulatory mechanisms. However, in leishmania and other related trypanosomatids, these responses appear to be accomplished mainly by post-transcriptional mechanisms [5]. In this setting, RNA-binding proteins (RBPs) are emerging as key regulatory factors that aid in post-transcriptional gene regulation. The shift from transcriptional to post-transcriptional control in these organisms appears to be due to an anomalous arrangement of trypanosomatid protein coding genes [6]. In addition to this specialized arrangement, the genes lack canonical promoters [7]. Transcriptional control of gene expression poses a challenge in these organisms as RNA polymerase II transcribes all protein encoding genes at comparable rates [8]. Nevertheless, the final output of mature mRNAs and proteins can vary tremendously even between adjacent genes. One post-transcriptional regulatory mechanism used by leishmania to control gene expression involves the processing of polycistronic pre-mRNAs into mature mRNAs. This RNA processing tool results in the trans-splicing of a capped spliced-leader exon at the 5’-end and a polyadenylation at the 3’-end [9, 10].

The association of RBPs with the 3’-UTRs of mRNAs usually leads to changes in stability, translation, or localization of target mRNAs, and this mechanism seems to be operative in trypanosomatids [11]. Over the past two decades, researchers have identified and characterized numerous RBPs in trypanosomatids, particularly *Trypanosoma cruzi* and *Trypanosoma brucei*. Recent advancements in sequencing and annotation of the trypanosomatid genomes have led to various predictions of novel RBPs [12, 13]. These predictions were based on finding classical mRNA structural motifs that serve as ligands for different RBPs [14]. Different transcripts carrying the same signature motifs are likely to be regulated in similar ways [15]. RBPs interact with mRNA motifs via unique functional domains such as RNA-Recognition Motifs (RRM), Zinc Finger domains (ZF) and Pumilio domains (PUM); and these motifs have been found in the RBPs of trypanosomatids [16]. For example, in silico screening of the genomes of *Trypanosoma brucei*, *Trypanosoma cruzi* and *Leishmania major* identified unique zinc finger proteins which are indicative of RNA binding [17]. In addition, computational analysis and bioinformatics have been used to predict and characterize RBPs of trypanosomes [15]. In vitro approaches have been used to pull down proteins bound to specific target 3’-UTR transcripts or, conversely, to pull down RNA transcripts bound to known RNA-binding proteins [18]. However, all of these approaches are limited and cannot provide a comprehensive picture of RBPs in the organisms of interest. Identification of the repertoire of trypanosomatid mRNA interacting proteins is essential for organizing our current molecular and genetic understanding of gene expression and in particular, post-transcriptional gene regulation. Accordingly, it was pertinent to develop an alternative strategy for characterizing RBPs in these pathogenic protozoa.

Recently, as part of an effort to identify mammalian *in vivo* mRNA-bound proteomes, three independent studies used a combination of UV RNA-protein cross-linking *in vivo* followed by Poly(A)-RNA pull downs [19–21]. This comprehensive approach is termed ‘‘interactome capture”, and these studies generated an atlas of mammalian mRNA-binding proteins *in vivo*. Recently, using a genome-wide tethering screen, in which random protein fragments were artificially bound to a reporter mRNA as bait, over 300 proteins were identified as possible mRNA-fate regulators by post-transcriptional mechanisms in *T*. *brucei* [22]. Since random protein fragments were artificially bound to a reporter mRNA, the results obtained may not be true for full-length proteins. Subsequently, the tethering screen was extended by the same group using a small-scale library of full-length open reading frames and this resulted in a catalogue of about 100 proteins that may regulate *T*. *brucei* mRNA-fate [23]. This same study also reported an mRNA-bound proteome consisting of 155 high-confidence RBPs from bloodstream forms of *T*. *brucei*. However, to the best of our knowledge no efforts have been reported to identify comprehensively the repertoire of RBPs in leishmania. We used *Leishmania donovani*, in its amastigote stage, as a paradigm for this study. We developed the interactome by combining UV cross-linking and oligo(dT) capture to pull down proteins bound to mRNAs in viable *L*. *donovani* amastigotes. We show that the *in vivo* leishmania mRNA interactome consists of at least 79 proteins, 49 of which show no significant homology to identified human RBPs in the database and we discuss resulting insights into the RNA biology of leishmania.

## Materials and Methods

### Parasite cultures

#### Leishmania promastigote culture

*L*. *donovani* Sudan strain S2 promastigotes were routinely cultured in M199 (Sigma-Aldrich) with 10% heat inactivated fetal bovine serum (FBS, Gibco), 20 mM HEPES (Sigma-Aldrich), 6 μg/mL hemin (Sigma-Aldrich), 10 μg/mL folic acid (Sigma-Aldrich), 2 mM L-glutamine (Stemcell), 100 U/mL penicillin/streptomycin (Sigma-Aldrich) and 100 μM adenosine (Sigma-Aldrich) at 26°C. Every 3 days the parasites were subcultured 1:10 in fresh medium and were kept in culture for a maximum of 20–25 passages. In order to maintain virulence and infectivity, fresh parasites were routinely obtained by purification of amastigotes from spleens of 6–8 week infected Syrian Golden hamsters followed by *in vitro* transformation into promastigotes by culturing for 5–7 days at 26°C in promastigote medium. Prior approval for animal experiments was obtained from the Animal Care Committee of University of British Columbia (approval# A14-00218)

#### Leishmania axenic amastigote culture

*In vitro* culture of amastigotes was carried out as follows: late logarithmic phase cultures of *L*. *donovani* promastigotes were transferred from promastigote medium at 26°C and pH 7.4 to RPMI 1640 medium supplemented with 10% fetal bovine serum and incubated at 37°C (5% CO_2_) for 16–18 h. Subsequently, cells were collected by centrifugation (1200×*g* at room temperature for 10 min) and resuspended in amastigote medium (RPMI 1640 with 25% fetal bovine serum, 100 μM adenosine and pH was titrated to pH 5.5 with 1M MES) at 37°C in 5% CO_2_ environment. Three days after initiation of transformation, cells were diluted in fresh amastigote medium and maintained by changing media every 4^th^ day.

### UV-cross linking of leishmania amastigotes

Dividing amastigotes (10 x 10^9^) were washed once with cold phosphate buffered saline (PBS) and resuspended in cold PBS to a concentration of 1.7 x 10^9^ per ml. The cell suspension (6 ml) was dispersed into a 100 mm x 20 mm cell culture plate without lid, placed on ice pack (immediately before cross-linking), and UV-irradiated 3 times (1 min each with 2 mins gap on ice) with 0.15 J cm− ^2^ at 254 nm using Ultra-LUM electronic ultraviolet cross linker at a distance of 15 cm from the UV source. After UV irradiation, cells were collected by centrifugation.

### Preparation of parasite protein extracts

Pellets from non irradiated control and irradiated amastigotes were lysed separately in 10 ml of ice cold stringent lysis buffer (20 mM Tris-HCl (pH 7.5), 500 mM LiCl, 0.5% lithium dodecyl sulfate, 1 mM EDTA and 5 mM DTT) containing 1 mM phenylmethylsulfonyl fluoride, 10 μg of aprotinin/ml, 10 μg of leupeptin/ml, and 2 μg of pepstatin A/ml. Pellets were resuspended by pipetting up and down. The suspended samples were then passed at least three times through a syringe with a narrow needle or until the homogenate became clear. The lysates were incubated for 10 min at 4°C.

### Oligo(dT) capture

300 μl of Oligo(dT)_25_ magnetic beads (New England Biolabs) per sample, previously equilibrated in three volumes of lysis buffer, was added to the lysate and incubated them for 1 h at 4°C with gentle end-over-end rotation. The tubes were placed on a magnet at 4°C for 5 mins to completely capture the magnetic beads. The beads were resuspended in 5 ml of ice-cold lysis buffer and washed for 5 mins at 4°C by gentle end-over-end rotation, and then pelleted with the help of a magnet. Recovered beads were resuspended in 5 ml of ice-cold wash buffer 1 ((20 mM Tris-HCl (pH 7.5), 500 mM LiCl, 0.1% LiDS, 1 mM EDTA and 5 mM DTT) and incubated for 5 mins at 4°C by gentle end-over-end rotation and beads were pelleted with the help of a magnet. 5 ml of ice-cold wash buffer 2 (20 mM Tris-HCl (pH 7.5), 500 mM LiCl and 1 mM EDTA) was added to pelleted beads which were resuspended and incubated for 5 mins at 4°C by gentle end-over-end rotation and beads were pelleted using a magnet. A final wash was given to pelleted beads with low salt buffer (20 mM Tris-HCl (pH 7.5), 200 mM LiCl and 1 mM EDTA). Elution of the mRNA-protein complexes was performed by incubating washed beads with 250 μl/ml no salt elution buffer (20 mM Tris-HCl (pH 7.5) and 1 mM EDTA) for 3 mins at 55°C. RNA concentration was determined using a Nanodrop device.

### RNA analysis

The RNA quality was assessed by Bioanalyzer chips (Agilent) using buffers and instructions provided by the manufacturer.

### RNA digestion and protein concentration

To recover mRNA bound proteins, mRNA-protein complexes were incubated with RNAse A (Fermentas) for 1h at 37°C. The RNAse-treated eluate was then concentrated by transfer to a 2-ml Amicon Ultra 10 3-kDa cutoff device and topped up with buffer 4 (20 mM Tris-HCl (pH 7.5) and 50 mM NaCl).

### Preparations of samples for mass spectrometry analysis

Resulting protein solutions were reduced and alkylated as per [24]. and digested with 1μg of trypsin (Promega) at 37°C overnight. Peptide samples were purified by solid phase extraction on C-18 STop And Go Extraction (STAGE) tips [25], and each treatment was chemically labeled by reductive dimethylation using formaldehyde isotopologues [26]. The labelled peptides were mixed and purified again by C-18 STAGE tips.

### HPLC and mass spectrometery anslysis

Final peptides were loaded onto quadrupole–time of flight mass spectrometer (Impact II; Bruker Daltonics) on-line coupled to an Easy nano LC 1000 HPLC (ThermoFisher Scientific) using a 40-50cm long analytical column packed with 1.9 μm-diameter Reprosil-Pur C-18-AQ beads (Dr. Maisch, www.Dr-Maisch.com), fixed on an in-house constructed column heater set at 50°C. Buffer A consisted of 0.1% aqueous formic acid, and buffer B consisted of 0.1% formic acid and 80% acetonitrile in water. Samples were resuspended in buffer A and loaded with the same buffer. Samples were analyzed over 90 min peptide separation and the column was washed with 100% Buffer B for 15 min, and re-equilibrated with Buffer A. The analysis was performed at 0.25 μl/min flow rate and the Impact II was set to acquire in a data-dependent auto-MS/MS mode with inactive focus fragmenting the 20 most abundant ions (one at the time at 18 Hz rate) after each full-range scan from m/z 200 Th to m/z 2000 Th (at 5 Hz rate). The isolation window for MS/MS was 2 to 3 Th depending on parent ion mass to charge ratio and the collision energy ranged from 23 to 65 eV depending on ion mass and charge. Parent ions were then excluded from MS/MS for the next 0.4 min and reconsidered if their intensity increased more than 5 times. Singly charged ions were excluded since in ESI mode peptides usually carry multiple charges. Strict active exclusion was applied. Mass accuracy: error of mass measurement is typically within 5 ppm and is not allowed to exceed 10 ppm. The nano ESI source was operated at 1700 V capillary voltage, 0.20 Bar nano buster pressure, 3 L/min drying gas and 150°C drying temperature.

### Mass spectrometry data analysis

Analysis of Mass Spectrometry Data was performed using MaxQuant 1.5.1.0. The search was performed against a database comprised of the protein sequences from Uniprot’s *Leishmania donovani* (bpk282a1) plus common contaminants using the following parameters: peptide mass accuracy 10 ppm; fragment mass accuracy 0.05 Da; trypsin enzyme specificity, fixed modifications—carbamidomethyl, variable modifications—methionine oxidation and N-acetyl protein. Only those peptides exceeding the individually calculated 99% confidence limit (as opposed to the average limit for the whole experiment) were considered as accurately identified.

### Data availability

The mass spectrometry proteomics data have been deposited to the ProteomeXchange Consortium via the PRIDE partner repository with the dataset identifier PXD005601 [27]

## Results and Discussion

### In vivo capture of leishmania amastigote mRNA binding proteins

**Fig 1:**
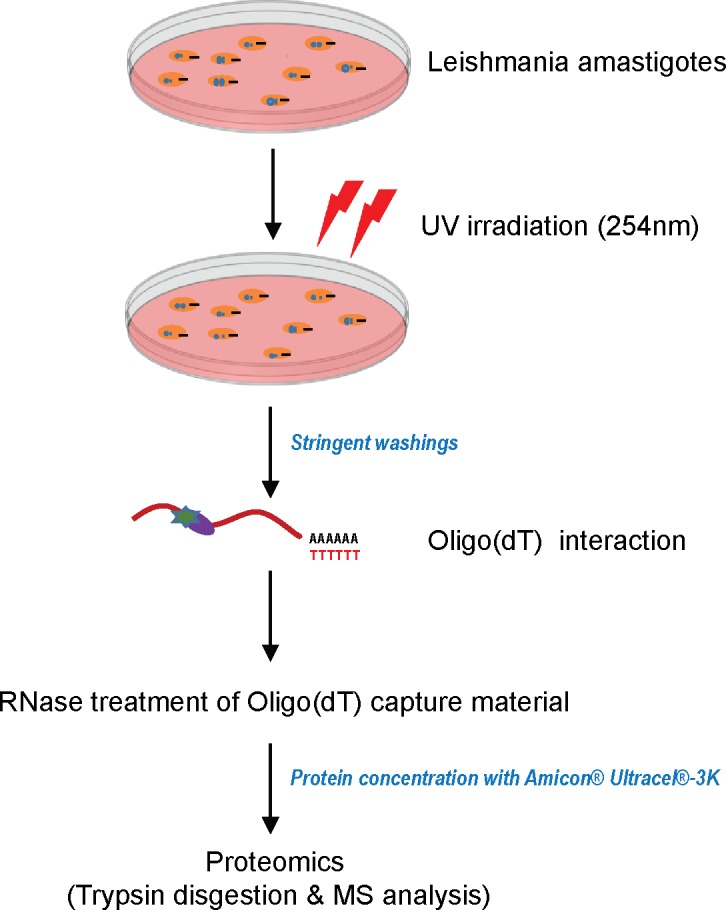
Schematic representation of *L*. *donovani* amastigotes interaction capture & mass spectrometry (MS) analysis. Live *L*. *donovani* amastigotes were UV irradiated to stabilize mRNA-protein interactions. These complexes were isolated by oligo(dT) magnetic beads capture and stringent washings. Bound protein-mRNA complexes were eluted with RNase treatment and identified by MS.

To characterize leishmania amastigote *in vivo* mRNA interacting proteins, we developed an approach based on UV irradiation of live amastigotes. UV irradiation generates highly reactive, short-lived states of the nucleotide bases within RNAs, resulting in covalent bond formation only with amino acids in direct contact. In order to covalently couple RBPs to mRNAs in a physiological *in vivo* state, dividing amastigotes were irradiated with UV light at 254 nm. In fact, it has been shown that UV light at 254 nm cross-links the naturally photoreactive nucleotide bases, especially pyrimidines, with amino acids such as Phe, Trp, Tyr, Cys, and Lys [28, 29]. Notably, this method offered several advantages over previous leishmania RBP identification methods: (i) UV irradiation induces covalent bond formation between nucleotide bases within RNAs only with amino acids in direct contact (zero distance] [30]; however, UV irradiation does not promote protein-protein cross-linking [30, 31], (ii) UV light was directly applied to living amastigotes, thus capturing physiological *in vivo* RNA-protein interactions, and (iii) mRNA poly(A) tail-oligo(dT) hybridization is very stable in high salt and ionic detergent containing buffers thus allowing efficient removal of non-specific interactions. In order to increase the efficiency of UV cross-linking, approximately 10 x 10^9^ leishmania amastigotes were used as starting material for each analysis. After UV irradiation of living amastigotes at 254 nm, cells were lysed in buffer containing high salt and ionic detergent. Following cell lysis, RBPs covalently bound to polyadenylated RNAs *in vivo* were captured on oligo(dT) magnetic beads. As a negative control, we included nonirradiated amastigotes cell lysates in the oligo(dT) capture assay. This nucleic acid hybridization based strategy allowed washings of affinity beads using highly stringent biochemical conditions including 500 mM lithium chloride and 0.5% lithium dodecyl sulfate. This protein-denaturing condition during the purification ensured the stringent isolation of proteins in direct contact with leishmania mRNAs through covalent bonds and allowed us to minimize contaminants. Following stringent washes, proteins were released from complexes by RNase treatment and were identified using MS (Fig 1). To test the efficiency of oligo(dT) pull-downs, we determined the amounts of beta-tubulin and glyceraldehyde 3-phosphate dehydrogenase (GAPDH) mRNAs in oligo(dt) pull-down material using RT-qPCR as compared with total RNA. There was a five to six fold enrichment in β-tublin and GAPDH mRNAS, in the oligo(dT) pull downs in comparison to the starting material (S1 Fig). This confirmed that the mRNAs were efficiently captured by the oligo(dT) affinity beads. Enrichment of mRNAs over rRNAs was independently confirmed using a Bioanalyzer Chip (S2 Fig). DNA did not copurify as no PCR amplification of beta-tubulin or GAPDH occurred when samples were RNase treated before the oligo(dT) pull-down or when reverse transcriptase (RT) was omitted from the RT reaction during complementary DNA (cDNA) preparation (data not shown).

### Identification of leishmania mRNA-bound proteome by mass spectrometry

To identify proteins cross-linked to mRNAs in UV irradiated leishmania, we performed oligo(dT) purifications followed by the release of bound proteins by RNAse treatment. The isolated proteins were concentrated using an Amicon Ultra 10 3-kDa device. The concentrated proteins were separated by SDS-PAGE followed by in-gel digestion with trypsin. Peptides were analyzed by nanoflow high-performance liquid chromatography and online mass spectrometry. By processing the data with MaxQuant 1.5.1.0, we selected proteins which were identified in proteomic analyses of three biological UV cross-linked replicates. In parallel, non-cross-linked mRNA bound samples were analyzed for specificity control. To perform statistical data analysis, protein enrichment in UV irradiated samples, as compared to non-irradiated samples, was assessed by considering intensity values. These values were derived by summing up all the extracted ion current of mass-to-charge values over time associated with the protein using MaxQuant [32, 33] and the results are presented in Supporting Information (SI) S1 Table. For statistical analysis we used one-tailed, paired t-test with the confidence interval of 90%. This analysis resulted in a list of 79 proteins found in three independent cross-linked samples (S1 Table). p-values were corrected using Benjamini-Hockberg procedure [34]. It is likely that the coverage of RBPs for the amastigote form of *L*. *donovani* is not complete. This protocol will not detect RBPs that are (i) not interacting with poly (A) RNAs; (ii) not expressed in amastigote form of *L*. *donovani*; (iii) not active under the experimental conditions; or (iv) not in sufficient quantity in the starting material used for the analysis. Nevertheless, the mRNA-bound proteome of the amastigote form of *L*. *donovani* is comprised of a complex mixture of proteins enriched in RNA-related activities (S2 Table).

### Overview of identified mRNA-interacting proteins

**Fig 2:**
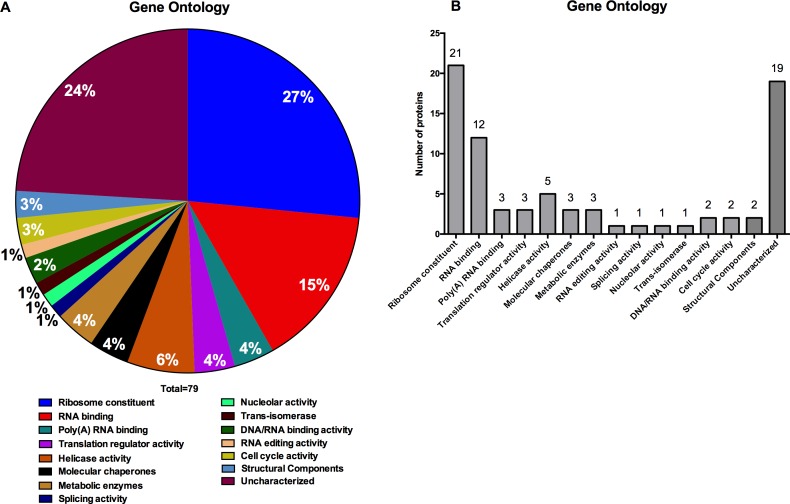
A and B. Gene Ontology enrichment analysis of *L*. *donovani* mRNA interacting proteins.

We first classified the identified proteins into functional categories based on gene annotation. As expected, ribosomal proteins, RNA helicases, translation factors, and classical RNA-binding proteins were most frequent, making up close to 70% of the identified proteins (S2 Table, Fig 2A & 2B). The low numbers of highly expressed cellular proteins such as metabolic enzymes suggested that the oligo(dT) purification was specific.

In addition to known mRNA-interacting proteins, we identified 19 proteins (S2 Table), that have not previously been annotated as RNA binding. Interestingly, 2 out of 19 seem to have RNA binding motifs (Protein ID: E9BH26 has 2 RNA-Recognition Motifs (RRMs) and protein ID: E9B8L4 has tryptophan-aspartic acid (WD) repeats regions (S2 Table). These findings suggest that the approach we used had the capability to identify RNA-interacting proteins that use distinct or highly divergent RNA-binding domains.

Some of the RBPs we identified are involved in protein translation. Elongation factor 1-alpha (S2 Table) is an abundant multifunctional protein. In addition to its canonical role in translation, it has also been involved in other cellular processes such as nuclear transport, cellular organization, protein quality control, co-translational degradation, mitochondrial tRNA import [35, 36] and macrophage SHP-1 activation [37]. Elongation factor-2 (S2 Table) is involved in translocation of the ribosome along the mRNA strand during translation [38, 39].

The *Leishmania* genome encodes three poly(A)-binding proteins and we were able to identify all three (Protein ID: E9BHB2, EBSU0 and E9BT55) (S2 Table). We also identified three proteins (Protein ID: E9BE15, E9BM42 and E9BTJ1) that are involved in energy metabolism. These were unexpected results, since these proteins do not possess known RNA binding domains in their structures. However, in yeast and other organisms, metabolic multifunctional enzymes capable of binding to RNA sequences have been previously reported [40, 41]. Indeed, an increasing number of multifunctional enzymes are being described in parasitic protozoa [42]. We identified five helicases (Protein ID: E9B8A4, E9B909, E9BNE6, E9BRR9 and E9BS1) (S2 Table) in the proteome of leishmania mRNA interacting proteins. DEAD-box RNA helicases mediate conformational change in RNA containing complexes and these changes are required to unwind RNA duplexes or disrupt RNA-protein interactions [43]. Two proteins -alpha tubulin and beta tubulin- bound to mRNAs which have no RNA-related gene ontology annotation. However, interestingly this unexpected interaction has been shown in recently published plant mRNA interactome [44]. Moreover, participation of the cytoskeleton in the spatial organization and regulation of translation has recently been suggested [45]. In addition, we also found three molecular chaperones (HSP-70, HSP-83-1 and mitochondrial HSP-60) (S2 Table). RNA-binding roles are not unprecedented for HSPs as mammalian HSP70 is able to bind to the 3’UTR of labile mRNAs [46]. In addition, it is known that human HSP-70 binds to and stabilizes endogenous AU-rich element-containing mRNAs [47]. Moreover, HSP90 is involved in RNA interference in human and in yeast [48]. It is also possible these HSPs are interacting nonspecifically due to their sticky nature.

It was of interest to ask whether any of the leishmania RBPs that we identified had previously been implicated in leishmania pathogenesis. In fact, we found that three of the RBPs (A0A0R4J963): Elongation factor 1-alpha [37], E9BTN1: Universal minicircle sequence binding protein [49] and E9BTJ1: Fructose-bisphosphate aldolase [50] are considered to potentially contribute to the pathogenesis of leishmania infection (S2 Table).

### mRNA binding domains of leishmania RBPs

**Fig 3:**
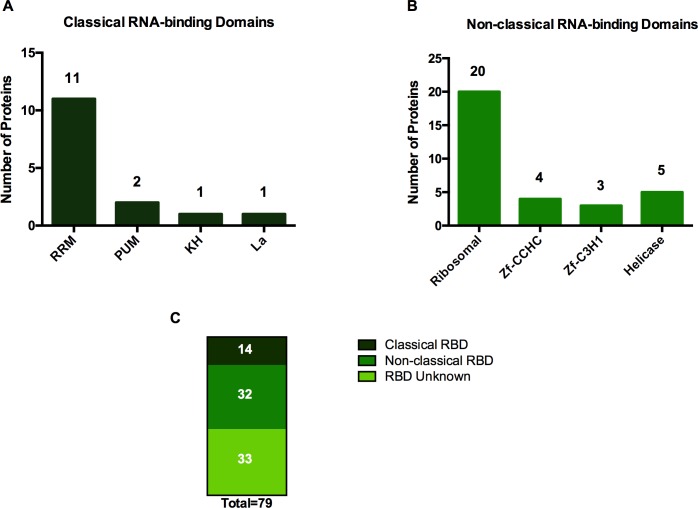
Globular domains in *L*. *donovani* mRNA interactome. Number of proteins harboring (A) classical and (B) non-classical RNA binding motifs in *L*. *donovani* mRNA interacting proteome. (C) Consolidated number of RNA binding motifs in 79 identified proteins, including 32 unknown RNA binding domains (RBD unknown).

To gain insights into modes of interaction of RBPs with mRNAs, the RBPs we recovered were analyzed for the presence of associated structures like functional domains (S2 Table). For this we performed Pfam and Inter-Pro domain enrichment analyses [14] which showed that most of the significantly enriched domains were various RNA-interaction motifs (Fig 3). In most eukaryotes, many RBPs interact with mRNAs via modular RNA binding domains (RBDs), including the RNA recognition motif (RRM), heterogeneous nuclear RNP K-homology domain (KH), zinc fingers (Znf) and others. [14]. These motifs have informed in silico algorithms to identify other proteins harboring similar signatures as putative novel RBPs [51]. However, numerous noncanonical RBDs have also been reported [52–55], reflecting limitations in the scope of computational predictions. Analysis of the leishmania interactome showed that about half of the mRNA interacting proteome contained known RBDs. This included several classical and nonclassical RBDs (Fig 3). The RNA-Recognition Motif (RRM) is one of the most commonly found protein domains in nature. Our analysis identified eleven RRM containing proteins (S2 Table, Fig 3). RRMs are most often involved in sequence-specific interactions with single-stranded RNAs. Proteins that contain RRMs are involved in all pathways of the RNA cycle, from splicing to mRNA turnover. RRMs are usually present in multiple copies within a protein and this multiplicity enhances ligand specificity [14].

Another commonly recognized RNA binding domain is the zinc finger domain. This domain was first described as a DNA-binding domain and was subsequently found in proteins that interact with RNA [56]. We found seven in our analysis, four containing CCHC type and three containing C3H1-type Zinc finger motifs (S2 Table, Fig 3). Besides the commonly recognized RNA-binding domains, we also found PUM and hnRNP K-homology (KH) RNA binding domains (Fig 3). The best characterized function of the PUM containing Pumilio protein is as a post-transcriptional repressor [57]. We found two PUM containing proteins in our analysis (S2 Table, Fig 3). hnRNP K-homology domain containing proteins are associated with multiple gene expression regulatory mechanisms, such as transcriptional regulation, and translational control [58]. We found only one hnRNP K-homology domain containing protein in the leishmania interactome (S2 Table, Fig 3).

### Comparison with previously known RBPs of leishmanial

To estimate the depth of the mRNA-bound proteome we captured in our oligo(dT) precipitations, we compared the number of identified proteins with previously known RBPs of leishmania. For this purpose, we began by examining the genome of *L*. *donovani* for potential RBPs. Recently, the whole genome of *L*. *donovani* strain bpk282A1 has been sequenced and annotated. Searching this genome for predicted proteins containing RNA binding domains from the complete predicted proteome of *L*. *donovani* resulted in 67 proteins. Interestingly, out of these 67 predicted RBPs, 13 were part of the *L*. *donovani* interactome reported here (S3 Table) providing experimental support for their *in silico* identification as putative RNA binding proteins.

Recently, *L*. *braziliensis HSP70* mRNAs interacting proteins have been reported [18]. We also compared our list of leishmania RBPs with RNA-binding domain containing proteins observed in this *in vitro* study to identify proteins that interact with *L*. *braziliensis HSP70* mRNAs [18]. In this study the 5’ UTR and the two types of 3’ UTRs from *L*. *braziliensis HSP70* genes were used as bait in pull down assays using total protein parasite extracts. The bound proteins were separated by two-dimensional gel electrophoresis and identified by MS. This study resulted in the identification of 52 different proteins based on their interaction with *L*. *braziliensis HSP70*-mRNAs [18]. Out of this list of 52 proteins, 12 were represented in our *in vivo* proteome of *L*. *donovani* amastigote mRNA interacting proteins (S3 Table). The differences in composition of our mRNA interacting proteome and the *HSP70* study could be due to *in vivo* versus *in vitro* mRNA interactions, different leishmania species or different washing conditions to capture bound proteins. Very recently, the mRNA-bound proteome of the bloodstream forms of *T*. *brucei* has been reported [23]. For this proteome, the authors performed UV-crosslinking of bloodstream forms of *T*. *brucei* followed by lysing of parasites and oligo (dt) capture of RBPs. Bound proteins were analyzed by mass spectrometry. This analysis resulted in the identification of 155 proteins highly enriched in RNA-related activities. Out of this list of 155 proteins, only 25 were represented in our *in vivo* proteome of *L*. *donovani* amastigotes mRNA interacting proteins (S3 Table). This low level of similarity (approximately 16%) between mRNA interactomes of *L*. *donovani* and *T*. *brucei* was a bit surprising as both parasites belong to family Trypanosomatidae. These differences in number and composition of mRNA interacting proteins could represent differences in their quite divergent life styles; *T*. *brucei* bloodstream being extracellular and leishmania amastigotes living inside phagolysosomes of mononuclear phagocytes with much reduced metabolic activities.

As discussed above, one group of RNA binding proteins is defined by the presence of a CCCH type zinc finger motif that directly binds to RNA [56]. Zinc finger proteins are RNA binding proteins with regulatory functions at all stages of mRNA metabolism. Genome-wide *in silico* screens for CCCH-type zinc finger proteins of *Trypanosoma brucei*, *Trypanosoma cruzi* and *Leishmania major* resulted in a list of CCCH-type finger proteins [16, 17]. Surprisingly, our proteome analysis did not identify any CCCH motif containing proteins. This lack of congruence could be due to species differences, low levels of expression in the amastigote stage of *L*. *donovani* or inactivity under the conditions of the experiment, such as not being efficiently cross-linked upon UV irradiation or possibly to false positive computational predictions. Despite the fact that our analysis did not identify any CCCH motif containing proteins, we were able to identify seven proteins containing C3H-1 type (4/7) and CCHC-type Zinc finger motifs (3/7) (Fig 3).

### Identification of mRBPs in the secretome of *L*. *donovani*

**Fig 4:**
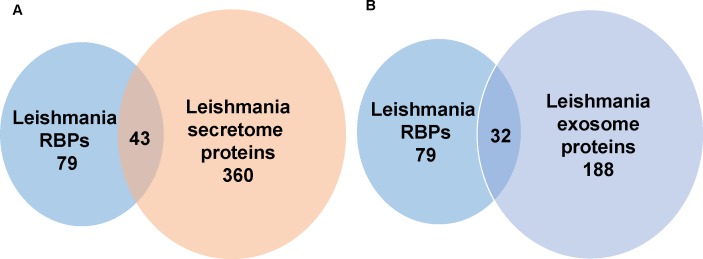
Detection of *L*. *donovani* mRNA interacting proteins in secretome. (A) Forty three *L*. *donovani* mRNA interacting proteins were detected in previously identified *L*. *donovani* secretome. (B) Thirty two *L*. *donovani* mRNA interacting proteins out of the above 43 proteins were detected in previously identified *L*. *donovani* exosome proteome.

Recently, we characterized the secretome of *L*. *donovani* and this led to the discovery of previously unrecognized exosome-based secretion pathway [59]. Using liquid chromatography-tandem mass spectrometry, we found that the leishmania secretome contained 360 proteins and 188 of them were detected in exosomes [59]. We also showed that leishmania exosomes are involved in the delivery of protein and RNA cargo to host macrophages to promote cell deactivation [60]. Functional annotation of the leishmania secretome revealed the presence of proteins involved in mRNA metabolism and synthesis [61]. It seems likely that the secretion of at least some of these leishmania RBPs may be part of the strategy used by leishmania to survive inside macrophages. One potential mechanism could be that leishmania secretome RBPs compete with host RBPs to skew host gene expression and create a phenotype which is permissive for infection. In fact, leishmania elongation factor 1 alpha and fructose-bisphosphate aldolase detected in the leishmania mRNA interactome (S2 Table) have previously been found in the cytosol of leishmania infected cells [37, 50] and may contribute to macrophage deactivation [37, 50]. Thus, it was of interest to ask how many other identified leishmania RBPs were represented in the leishmania secretome. Notably, from the list of 360 proteins in the leishmania secretome, 43 were represented in our *in vivo* proteome of *L*. *donovani* mRNA interacting proteins (S4 Table, Fig 4 panel A). Remarkably, 32/43 of these proteins were exclusively present in the exosome portion of the secretome (S4 Table, Fig 4 panel B).

### Human orthologs

In order to search for human orthologs for the leishmania mRNA interacting proteins we identified, BLAST search was performed against the human RBPs database (http://rbpdb.ccbr.utoronto.ca/). The protein list presented in S2 Table shows 32 human orthologs. The remaining 47 leishmania mRNA interacting proteins showed no significant homology to identified human RBPs in the database. This list of 47 leishmania specific mRNA interacting proteins is of particular interest as these proteins are potential leishmania drug targets.

In summary, the findings presented herein identify for the first time the *in vivo* mRNA bound proteome of living leishmania amastigotes. This work presents a novel picture of the RNA biology of the life cycle stage of *L*. *donovani* responsible for causing visceral leishmaniasis in humans. The approach of interactome capture can also be applied to study changes in interactome composition as a function of various biological contexts, such as metabolic changes, low pH environment, oxidative stress, nutrient deprivation or response to drugs. Notably, this study identified a novel group of leishmania RBPs with no orthologs or homologs in the human genome and these may represent high quality candidates for selective drug targeting leading to novel therapeutics.

## Supporting Information

S1 Table *L*. *donovani* mRNA binding proteins with Max Quant intensity values in non-crosslinked and UV-crosslinked conditions. (XLSX)

S2 Table *L*. *donovani* mRNA binding proteins categorization. (XLSX)

S3 Table Comparison of our RBPS with published trypanosomatid RBPs. (XLSX)

S4 Table *L*. *donovani* mRNA interacting proteins identified in previously published *L*. *donovani* secretome/exosomes. (XLSX)

S1 Fig Levels of β-tubulin and GAPDH RNAs in oligo(dT) captured material. Equal amounts of RNA from oligo(dt) captured material and from starting input RNA were analyzed for the levels of GAPDH and β-tubulin RNAs by RT-qPCR (n = 3;±s.d).
(TIF)

S2 Fig Agilent Bioanalyzer RNA profiles of oligo(dT) captured RNA alongside input RNA. Equal amounts of input RNAs and oligo (dT) beads captured RNAs were analyzed using Agilent 2100 Bioanalyzer. The middle three prominent peaks in each panel (A-D) represent leishmania ribosomal RNAs.
(TIF)
